# Quantification of Oseltamivir Phosphate Enantiomeric Impurity by Chiral HPLC Method With the Aid of Solvent Extraction and Phosphate Salt‐Out Method

**DOI:** 10.1002/chir.70034

**Published:** 2025-05-07

**Authors:** Torati Srinivas, K. V. N. Suresh Reddy, Challa Madhavi, M. Kiranmai Reddy

**Affiliations:** ^1^ Department of Chemistry, GITAM School of Science GITAM (Deemed to be University) Visakhapatnam AP India; ^2^ Department of Life Sciences, GITAM School of Science GITAM (Deemed to be University) Visakhapatnam AP India

**Keywords:** enantiomeric impurity (3S, 4S, 5R), oseltamivir phosphate, salt‐out process, solvent extraction

## Abstract

A robust chiral high‐performance liquid chromatography (HPLC) method was established to separate and quantify the enantiomeric impurity (3S, 4S, 5R) in oseltamivir phosphate. A new sample preparation approach was used, involving the solvent extraction method to remove phosphate salt from the drug, thereby preventing the column's clogging and confirming method repeatability. Thin‐layer chromatography (TLC) was employed to identify oseltamivir in the organic layer, while ^31^P nuclear magnetic resonance (NMR) spectroscopy and the molybdenum blue method were used to confirm the presence of phosphate in the aqueous layer. An ion‐pair reversed‐phase HPLC method with indirect UV detection was utilized to quantify the phosphate in both the organic and aqueous phases. Chromatographic separation of enantiomeric impurity (3S, 4S, 5R) from oseltamivir phosphate drug substances (3R, 4R, 5S) was accomplished using a Chiralpak IC‐3 column with a mobile phase consisting of n‐hexane, methanol, isopropyl alcohol, and diethyl amine (85:10:5:0.2, *v/v/v/v*) at a flow rate of 0.6 mL/min and a detection wavelength of 225 nm. The selectivity of method is clearly proved by separating the impurity from oseltamivir phosphate drug substance, with a resolution of more than 3.0. The method displayed exceptional linearity over a range of 0.035–0.300%*w/w*, with limits of detection of 0.005%*w/w* and quantification of 0.035%*w/w*. Consistent recovery rates were obtained between 91% and 94%, and the analytical solution remains stable for up to 72 h at 2°C–8°C.

## Graphical abstract

In this method, oseltamivir phosphate enantiomeric impurity is quantified through the chiral HPLC method by removal of phosphate salt through solvent extraction coupled with the salt‐out method. Chiral HPLC confirms the presence of drugs and impurities in the organic layer. Ion‐pair HPLC confirms the presence of phosphate in the aqueous layer.

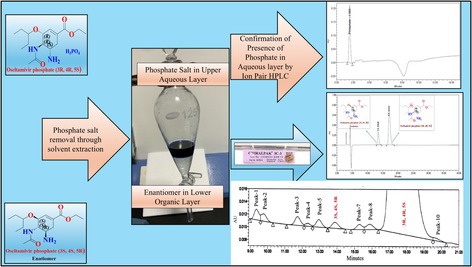

## 1 Introduction

Oseltamivir phosphate is used to prevent influenza (flu) [1], a highly contagious respiratory disease caused mainly by influenza types A and B. This flue easily spreads through droplets released when an affected person coughs or sneezes, causing a global health burden [2]. In 1999, the FDA (Food and Drug Administration) approved oseltamivir phosphate as the first oral therapeutic inhibitor for influenza neuraminidase, a crucial viral enzyme. It is commercially marketed as Tamiflu; oseltamivir phosphate is a prodrug that experiences conversion in the body to its active form, oseltamivir carboxylate, which reduces viral replication. The IUPAC name of oseltamivir phosphate is ethyl (3R, 4R, 5S)‐4‐acetamido‐5‐amino‐3‐pentan‐3‐yloxycyclohexene‐1‐carboxylate with a molecular weight of 312.4 g/mol bearing a formula of C_16_H_28_N_2_O_4_. It is partially insoluble in nonpolar solvents like methylene chloride but promptly dissolves in water and methanol [3].

**FIGURE 1:**
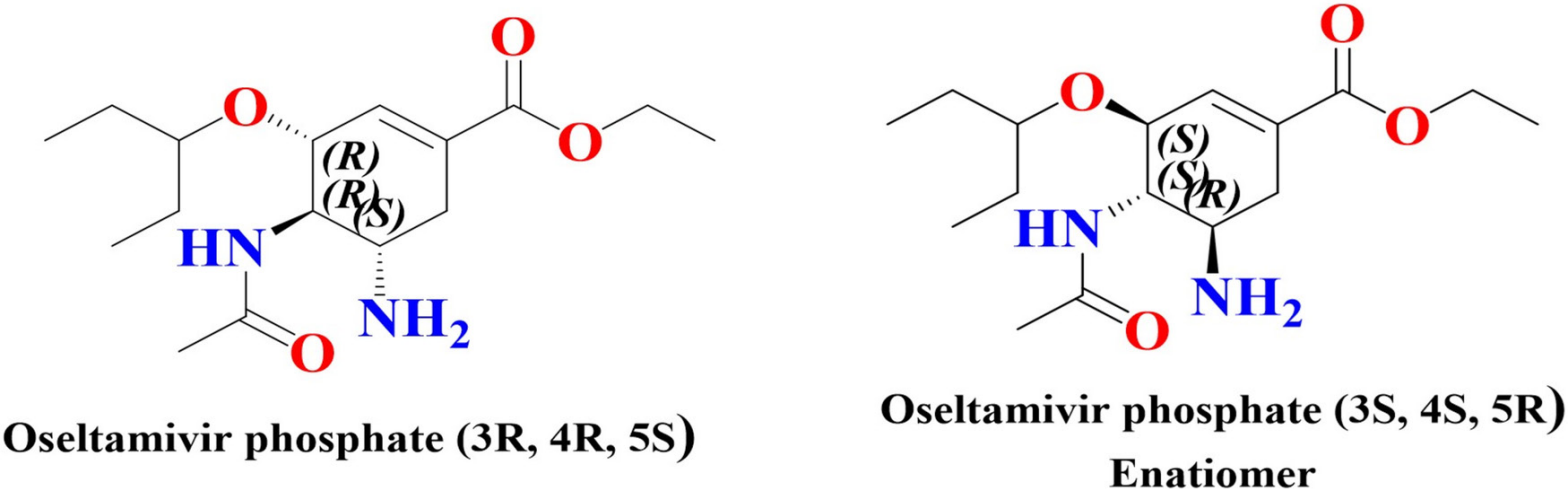
Chemical structures of oseltamivir phosphate (3R, 4R, 5S) and enantiomeric impurity (3S, 4S, 5R).

The enantiomeric impurity (3S, 4S, 5R) of oseltamivir phosphate and oseltamivir phosphate drug substance (3R, 4R, 5S) structure is shown in Figure 1. Oseltamivir phosphate structure contains a cyclohexene ring containing three stereogenic centers, primarily C3, C4, and C5, 3‐pentoxy group binds C3, an acetamine binds C4, and an NH_2_ group binds C5 and total of eight stereoisomers [4, 5, 6]. These chiral centers substantially influence the molecule's pharmacological activity. The studies indicate that enantiomers of chiral drugs exhibit distinct pharmacokinetic and pharmacodynamic properties, while one enantiomer interaction efficiently targets receptors and provides a therapeutic effect, and the opposite enantiomer might have reduced activity or could be affected by interaction with inadvertent receptors [7]. This phenomenon highlights the significance of chiral analysis and enantiomeric purity of the drugs. The International Council for Harmonization (ICH) and other regulatory bodies emphasize the need to confirm that enantiomeric impurities should be below certain thresholds [8, 9, 10].

Regardless of the serious requirement, enantiomeric analysis of oseltamivir phosphate poses significant challenges. The structure of enantiomers and other active pharmaceutical ingredients (APIs) is chemically identical, setting hurdles in their quantification and separation [11]. Moreover, the presence of phosphate salts in oseltamivir phosphate further aggravates the issue. This “memory effect” from phosphate salt accumulation compromises the method's accuracy, reproducibility, and robustness [12, 13].

An inclusive evaluation of the literature reveals numerous high‐performance liquid chromatography (HPLC) methods for analyzing oseltamivir phosphate in drug substances, drug combinations, and dosage forms. However, no standard methods are available to quantify the enantiomer impurity in the presence of phosphate salts [14, 15, 16, 17, 18]. Bequeathed the cumulative demand for enantiomeric purity in pharmaceuticals, this gap emphasizes the need for innovative solutions that combine chiral analysis and impurity control, particularly in phosphate salt–containing drug substances. So, this study provides an innovative approach combining normal phase chiral HPLC and solvent extraction to separate and quantify the enantiomeric impurity. This developed procedure uses a solvent extraction process with different solvents such as dichloromethane, sodium hydroxide, and sodium chloride to remove phosphate salts efficiently [19, 20, 21]. Extraction of phosphate into aqueous layer was confirmed by thin‐layer chromatography (TLC), molybdenum blue method [22], ^31^P nuclear magnetic resonance (^31^P NMR) spectroscopy [23], and ion‐pair reversed‐phase HPLC method with indirect UV detection technique [24]. This extraction method reduces phosphate salt deposition in the column, enabling reproducibility, improving peak shapes, and extending column lifespan.

The method has been validated as per ICH Q2 (R2) [25] parameters such as accuracy, precision, specificity, linearity, robustness, and ruggedness, facilitating its reliability for routine quality control monitoring in pharmaceutical manufacturing. This work fills the important gap in the literature by preparing the first detailed account of the chiral HPLC method for the quantitative analysis of the enantiomeric impurity (3S, 4S, 5R) of oseltamivir phosphate. The procedure's new extraction style solves the reproducibility issues connected with phosphate salts and improves compliance with regulatory standards.

## 2 Materials and Methods

### 2.1 Chemicals and Reagents

The oseltamivir phosphate drug substance, enantiomeric impurity, and other impurities were obtained from Tyndal Labs Pvt. Ltd., Visakhapatnam. The analytical grade reagents and solvents were used in this study. The chemicals such as n‐hexane, methanol, isopropyl alcohol, diethylamine, sodium hydroxide, dichloromethane, sodium chloride, sulfuric acid, ammonium molybdate, and ascorbic acid were procured from Merck India. Deuterium oxide (D_2_O) and deuterated chloroform (CDCl_3_) (NMR grade) were procured from Sigma Aldrich Isotope Laboratories Inc., with a deuterium content of 99.9%. Disodium hydrogen phosphate dihydrate, acetonitrile, tetrabutylammonium hydroxide, and phthalate were procured from Merck India. Ultrapure water has been acquired from Milli‐Q system from Millipore, which was used for all analytical methods.

### 2.2 Instrumentation and Software Chromatographic Conditions

The HPLC method used in this work was a Waters Technologies e2695 model equipped with a quaternary pump, degasser, column oven, autosampler, and a photodiode array (PDA) detector (Model 2998). The responses from the detector were monitored and assessed using Empower‐3 software. Weight measurements were accomplished using a RADWAG analytical balance (Model AS 82/220.R2 PLUS). A Bruker 500 MHz NMR instrument with Top Spin 4.3.0 software was used in this study.

### 2.3 HPLC Conditions for Enantiomeric Impurity

By using the chiral stationary phase (CSP), the chromatographic conditions were optimized; Chiralpak‐IC (150 × 4.6 mm, 3 μm) with cellulose tris (3,5‐dichlorophenylcarbamate) as the chiral selector was used for separation of enantiomeric impurity from oseltamivir drug substance. The mobile phase contains a premixed solution of n‐hexane, methanol, isopropyl alcohol, and diethylamine in a ratio of 85:10:5:0.2 (*v/v/v/v*). This mixture was pumped through the column at a 0.6 mL/min flow rate. The sample temperature was maintained at 5°C, while the column oven was set to 35°C. Detection was performed at a wavelength of 225 nm, with an injection volume of 10 μL and a total run time of 40 min.

### 2.4 Solution Preparation for the Analysis of Enantiomeric Impurity by Chiral HPLC

#### 2.4.1 Preparation of Sample Solution Without Extraction

About 30 mg of the test sample was accurately weighed and transferred into a 10‐mL volumetric flask; then, it was dissolved in methanol and diluted to the mark and mixed well.

#### 2.4.2 Preparation of 1.0 N Sodium Hydroxide Solution

An accurate amount of 20 g of sodium hydroxide was weighed and transferred into a 500‐mL volumetric flask. The contents were sonicated to aid dissolution, and Milli‐Q water was added to make up the final volume. The flask was then thoroughly mixed to ensure uniform distribution of the solution.

#### 2.4.3 Preparation of Blank Solution

A mixture of 10 mL of dichloromethane, 8 mL of 1 N NaOH solution, and 3.0 g of sodium chloride was transferred into a 125‐mL separation funnel. The funnel was shaken well and on standby for 2 min for complete layer separation of organic and aqueous layers and collected the lower organic layer. Subsequently, 1.5 mL of the collected solution (lower organic layer) was transferred into a 5‐mL volumetric flask, diluted to the mark with methanol, and mixed thoroughly.

#### 2.4.4 Preparation of Standard Solution

At about 1.2 mg of (3S, 4S, 5R) oseltamivir HCl (enantiomer) was accurately weighed and transferred into a 125‐mL separation funnel. To this, 10 mL of dichloromethane, 8 mL of 1 N NaOH solution, and 3.0 g of sodium chloride were added. The funnel was shaken well and on standby for 2 min which allowed to confirm complete layer separation. The lower organic layer was then collected. Consequently, 0.5 mL of the collected organic layer solution was accurately transferred into a 10‐mL volumetric flask diluted to the mark with methanol and mixed well.

#### 2.4.5 Preparation of Sample Solution

About 100 mg of the test sample was accurately weighed and transferred into a 125‐mL separation funnel; 10 mL dichloromethane, 8 mL of 1 N NaOH solution, and 3.0 g of sodium chloride were added. The mixture was mixed well for 2 min and allowed for complete separation of the upper aqueous layer and lower organic layer. Further, 1.5 mL of each layer was collected in two different 5‐mL volumetric flasks, diluted to the mark with methanol, and mixed well. The organic and aqueous layers that were obtained here were further used in the ion‐pair reversed‐phase HPLC, NMR, and molybdenum blue test studies for the phosphate content.

### 2.5 HPLC Conditions for Phosphate Content

Phosphate content in organic and aqueous layers was quantified as per the method described in the literature [24]. ad Hoc Alpha‐C18 stationary phase (150 × 4.6 mm), 3 μm, was used for phosphate content in aqueous and organic layer of oseltamivir drug substance. The mobile phase contains mixture of pH 8.2 buffer (containing 0.5 mM tetrabutylammonium hydroxide and 1 mM phthalate) and acetonitrile (95:5, %*v/v)*. This mixture was pumped through the column at a 1.0 mL/min flow rate. The sample temperature was maintained at 25°C, while the column oven was set to 25°C. Detection was performed at a wavelength of 254 nm, with an injection volume of 50 μL and a total run time of 15 min.

#### 2.5.1 Preparation of Phosphate Standard

About 76 mg of disodium hydrogen phosphate dihydrate was accurately weighed and transferred into a 50‐mL volumetric flask; then, it was dissolved in water, mixed well, and diluted to the mark.

### 2.6 Sample Preparation for ^31^P NMR Analysis

Of the organic layer, 0.1 mL was mixed with 0.4 mL of CDCl_3_, and 0.1 mL of the aqueous layer was mixed with 0.4 mL of D_2_O for ^31^P NMR analysis.

### 2.7 Solution Preparation for the Molybdenum Blue Test Method

#### 2.7.1 Preparation of Ammonium Molybdate Solution

Twenty grams of ammonium molybdate was weighed and transferred into a 100‐mL volumetric flask; then, it was dissolved in water and diluted to the mark and mixed well.

#### 2.7.2 Preparation of Ascorbic Acid Solution

A quantity of 1.76 g of ascorbic acid was weighed and transferred into a 100‐mL volumetric flask. It is dissolved and diluted to volume up to the mark with water and mixed well.

#### 2.7.3 Phosphate Identification Test Procedure

The separated organic and aqueous layers were placed in two 50‐mL test tubes. Each test tube was filled with 2.5 mL of ascorbic acid solution and 0.5 mL of ammonium molybdate solution to quantify phosphate.

## 3 Results and Discussion

**FIGURE 2:**
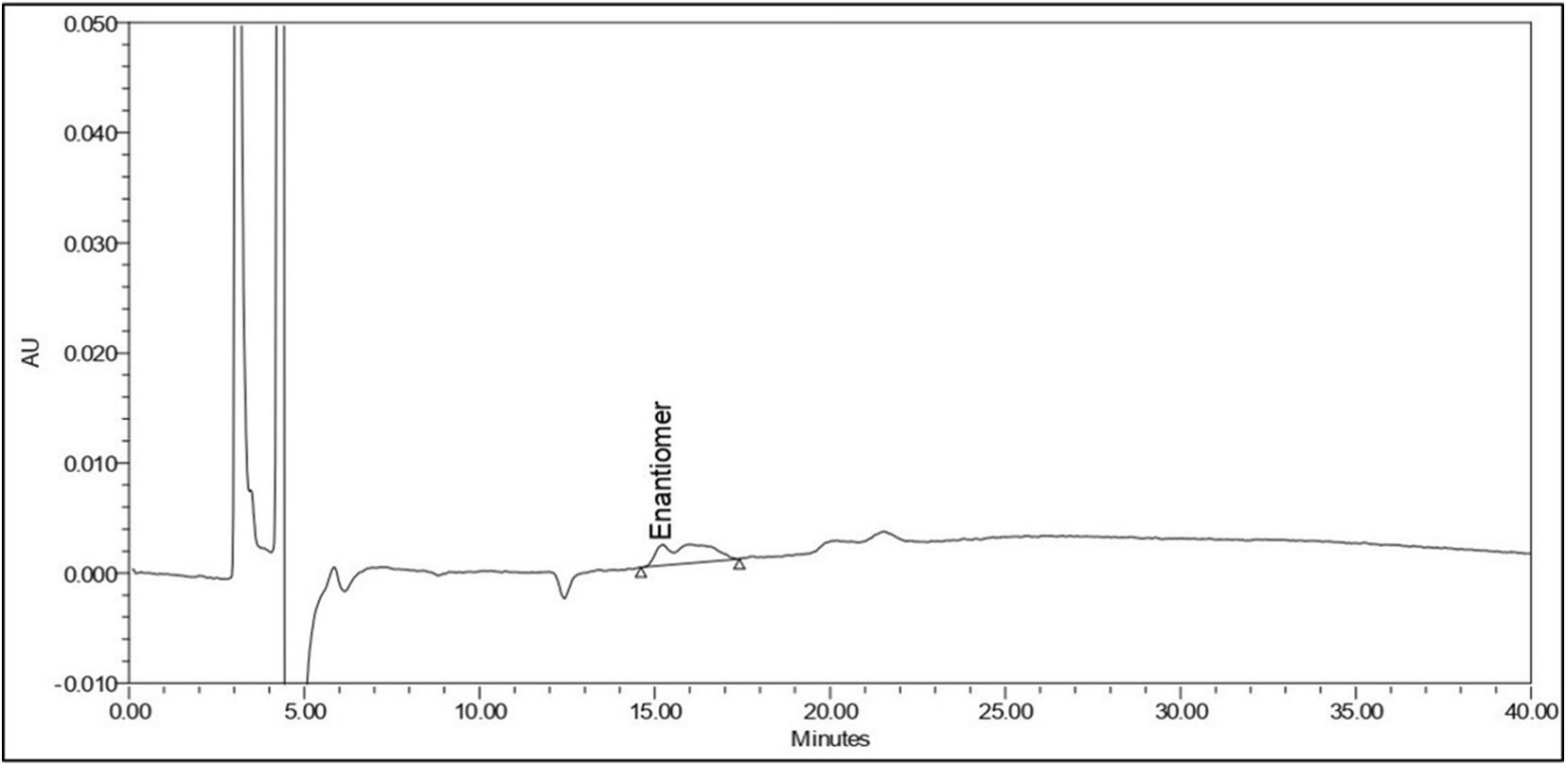
Oseltamivir phosphate enantiomeric impurity (3S, 4S, 5R) standard injection without phosphate extraction after two sample injections. Enantiomer concentration is 0.2%. Analytical column: Chiralpak IC (150 × 4.6) mm, 3 μm; mobile phase: n‐hexane, methanol, isopropyl alcohol, and diethylamine in 85:10:5:0.2 (*v/v/v/v*); flow rate: 0.6 mL/min; run time: 40 min; column temperature: 35°C; injection volume: 10 μL; UV detection at 225 nm.

**FIGURE 3:**
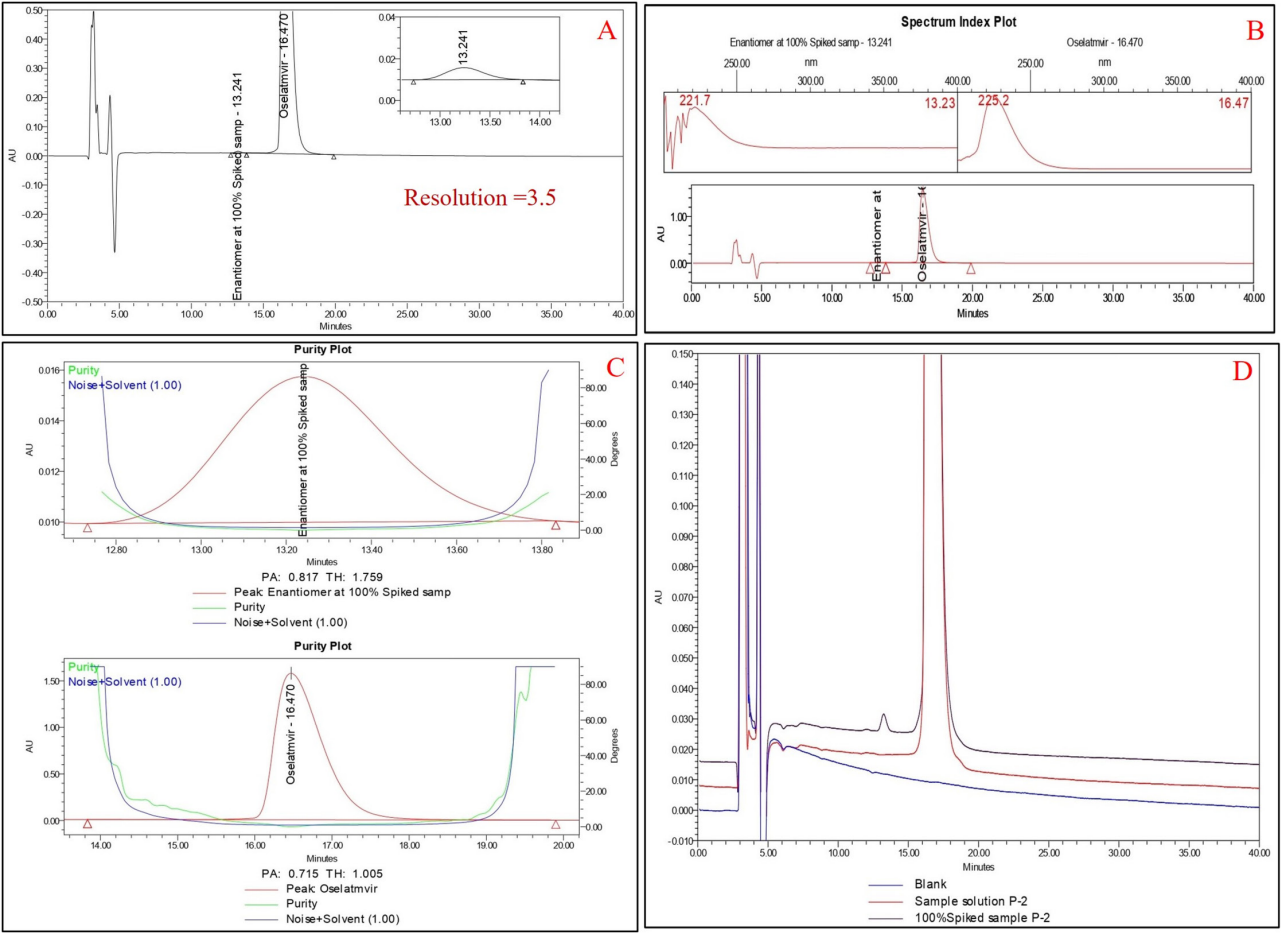
(A) Spiked sample solution. (B) PDA spectrum index plot. (C) Purity plot. (D) Overlay chromatogram of blank, oseltamivir phosphate (3R, 4R, 5S), and enantiomeric impurity (3S, 4S, 5R). Enantiomer concentration is 0.2%. Analytical column: Chiralpak IC, (150 × 4.6 mm), 3 μm; mobile phase: n‐hexane, methanol, isopropyl alcohol, and diethylamine in 85:10:5:0.2 (*v/v/v/v*); flow rate: 0.6 mL/min; run time: 40 min; column temperature: 35°C; injection volume: 10 μL; UV detection at 225 nm.

To accomplish the separation of oseltamivir phosphate enantiomeric impurity from the drug substance, various CSPs, including cellulose and amylose‐based derivatives, were evaluated, with carefully optimized mobile phase compositions. The separation process depends on chiral discrimination, which occurs when enantiomers interact with the stationary phase in different ways to form temporary complexes. These interactions are influenced by the structural characteristics of the CSPs, like rigid structure (cellulose‐based CSP) or helical structure (amylose‐based CSP), and molecular interactions such as hydrogen bonding, dipole–dipole forces, and π–π interactions. The attempts to separate the enantiomeric impurity using reversed‐phase mode were unsuccessful due to the low affinity of the enantiomers for the CSP or the trouble of inclusion the analyte into the chiral cavity. Moreover, the presence of phosphate salts in oseltamivir phosphate further aggravates the issue. Phosphate salts are insoluble in nonpolar solvents and can precipitate in chiral columns in normal phase mode, give irregular peak shapes (Figure 2), and lead to less repeatability and decreased column life span. The normal‐phase mode proved much more effective, extending high repeatability and clear separation. The use of a cellulose carbamate–derived chiral column (Chiralpak IC (150 × 4.6 mm), 3 μm) was found to be a successful separation of the enantiomers. The mobile phase was a premixed solution of n‐hexane, methanol, isopropyl alcohol, and diethylamine in the ratio of 85:10:5:0.2 (*v/v/v/v*) with a constant column temperature of 35°C and a flow rate of 0.6 mL/min detecting in a wavelength of 225 nm. The results showed that the resolution between the two enantiomers exceeded 3.0, demonstrating a clear and effective separation. Chromatograms for the blank, test, and spiked samples confirmed the method's reliability, and the chromatograms of precision study are displayed in Figure 3. This method successfully addressed earlier challenges, proving a strong and repeatable approach for analyzing enantiomer impurity in oseltamivir phosphate.

### 3.1 Method Development and Optimization

This study's primary concern is the quantitative analysis of the enantiomeric impurity in oseltamivir phosphate using HPLC. The chromatographic conditions were fine‐tuned to confirm the efficient resolution of the enantiomeric impurity, achieving precise retention times and peak symmetry. The proposed separation techniques have shown high specificity, confirming clear resolution of the enantiomeric impurity from drug substance and other impurities, including stereoisomers. Moreover, the extraction procedure was refined to provide reproducible results, minimizing matrix interference and enhancing sensitivity.

#### 3.1.1 Selection of Column, Organic Modifier, and Additive Modifier

Initially, in the reversed‐phase HPLC process, ammonium bicarbonate and dipotassium hydrogen phosphate solutions were used as buffers, and acetonitrile and methanol were used as organic modifiers in different ratios using Chiralpak IC, and Chiralpak IE columns in different isocratic and gradient programs, but enantiomeric peaks were not separated (Figure S1). When the X‐Bridge C8 column (250 × 4.6 mm, 5 μm) was used, all diastereomer peaks were successfully separated from the oseltamivir peak as shown in Figure S2, but enantiomeric impurity (3S, 4S, 5R) could not be separated from oseltamivir.

Subsequently, normal‐phase chiral conditions were adopted instead of reversed‐phase chiral chromatography for better separation. In the initial attempts at normal phase mode, the Chiralpak IC column and additives such as ethanolamine, triethylamine, and diethylamine were used. Different combinations of solvents, such as n‐hexane, ethanol, methanol, and isopropyl alcohol in various compositions, were used as mobile phases and found the broad peak shapes. Table 1 summarizes the different method development trials, and a few of the development trial chromatograms are displayed in Figures S3–S5. These trials showed broad peak shapes, no identification of the enantiomeric impurity, and no separation of the impurity from the drug substance. Ideal separation of enantiomer impurity (3S, 4S, 5R) from oseltamivir (3R, 4R, 5S) was achieved using a mobile phase comprising n‐hexane, methanol, isopropyl alcohol, and diethylamine in the ratio of 85:10:5:0.2 (*v/v/v/*v) at a flow rate of 0.6 mL/min using the Chiralpak IC column (150 × 4.6 mm, 3 μm) under isocratic conditions as shown Figure 3A. This procedure successfully separated the oseltamivir enantiomeric impurity (3S, 4S, 5R) from the oseltamivir peak (3R, 4R, 5S), with the enantiomeric impurity eluting at a retention time of 13.2 min. The resolution between the two peaks is more than 3.0, as displayed in Figure 3A.

**TABLE 1:** Method development trails by using Chiralpak IC‐3 column.

Mobile phase compositions	Proportion ratio (*v/v/v/v*)	Resolution
n‐Hexane:ethanol:methanol:ethanolamine	90:6:4:0.2	0.8
n‐Hexane:methanol:2‐propanol:diethyl amine	80:15:5:0.2	1.4
n‐Hexane:methanol:2‐propanol:diethyl amine	80:15:5:0.1	1.4
n‐Hexane:methanol:2‐propanol:diethyl amine	90:6:4:0.1	1.3
n‐Hexane:methanol:2‐propanol:diethyl amine	90:6:4:0.3	1.3
n‐Hexane:ethanol:methanol:diethyl amine	90:6:4:0.1	1.0
n‐Hexane:methanol:2‐propanol:diethyl amine	90:5:5:0.2	1.4
n‐Hexane:methanol:2‐propanol:triethlamine	85:10:5:0.2	—
n‐Hexane:methanol:2‐propanol:ethanolamine	85:10:5:0.2	—
n‐Hexane:methanol:2‐propanol:diethyl amine	85:10:5:0.2	3.0

#### 3.1.2 Selection of Diluent

Methanol was selected as a diluent due to its ability to dissolve oseltamivir phosphate, its enantiomeric impurity, and its miscibility with the extraction solvent (dichloromethane). Additionally, the enantiomeric impurity of oseltamivir phosphate exhibited a distinct peak shape when methanol was used as a diluent. Here, the test concentration of oseltamivir phosphate was maintained at 3 mg/mL.

#### 3.1.3 Column Oven and Sample Temperatures and Injection Volume

The selected injection volume of 10 μL resulted in a good response and higher sensitivity for the enantiomeric impurity. The column oven temperature (35°C ± 5°C) was varied to check the efficiency of separation of enantiomeric impurity from oseltamivir. The temperature changes did not significantly impact the peak elution and peak response. The sample temperature was maintained at 5°C.

### 3.2 Sample Treatment (Optimization of Solvent Extraction for Extraction of Phosphate Salt)

**FIGURE 4:**
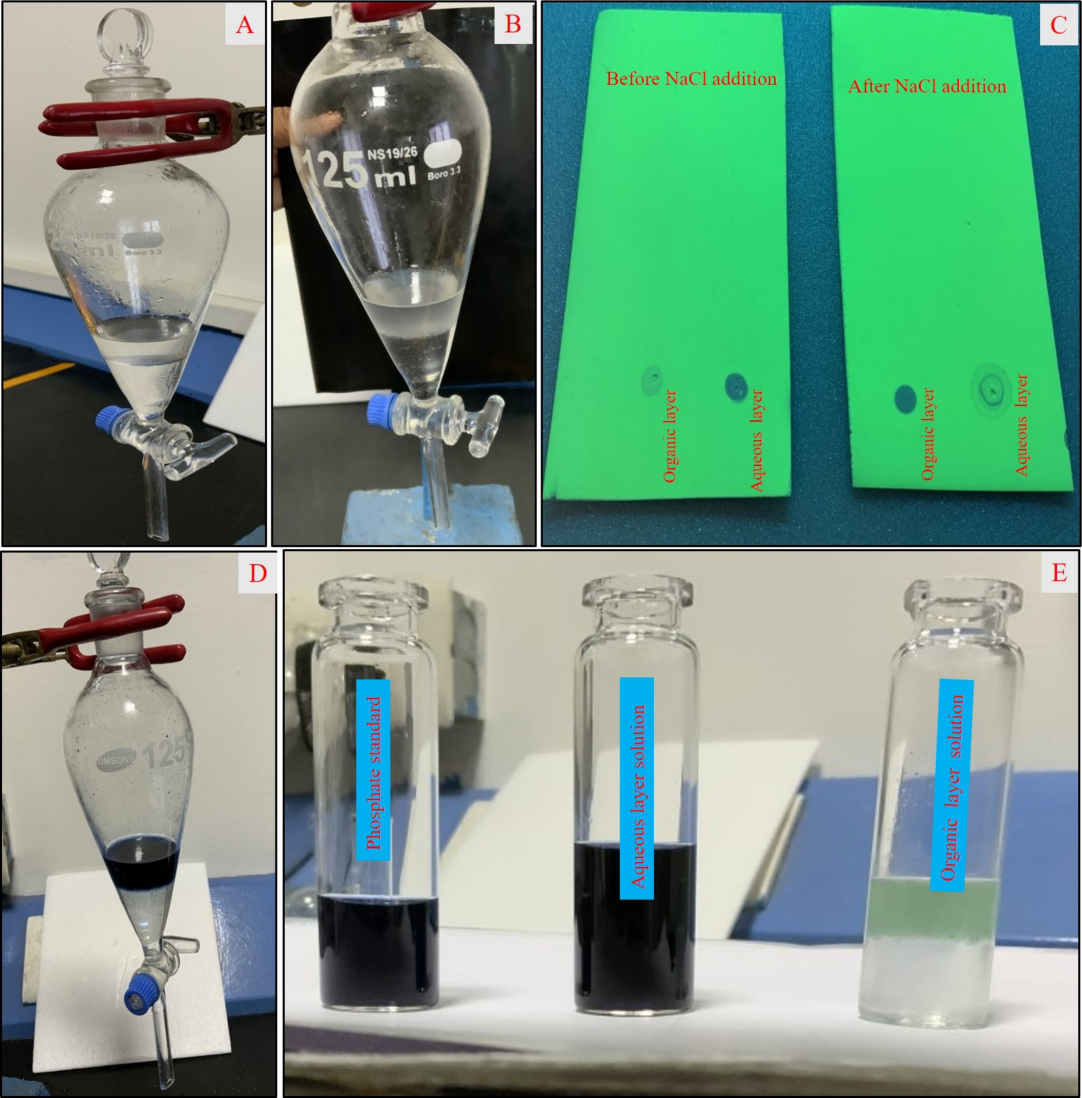
(A) Image of separating funnel contains dichloromethane and sodium hydroxide layers. (B) Image of separating funnel contains dichloromethane and sodium hydroxide layers after saturating with NaCl. (C) TLC images before and after addition of NaCl. (D) Image of separating funnel after the molybdenum blue test. (E) Images of standard phosphate solution, organic layer, and aqueous layer after the molybdenum blue test.

In the normal phase mode, phosphate salts are deposited into the chiral column when the sample passes into the stationary as they are insoluble in nonpolar solvents, leading to issues with repeatability and asymmetrical peak shapes. In order to remove phosphate, the solvent extraction method is chosen. The solvent used in this process should be chemically nonreactive and not miscible with aqueous solution. Due to miscibility of methanol (diluent) with sodium hydroxide, phosphate salt cannot be separated. First, n‐hexane, n‐heptane, cyclohexane, and dichloromethane were used as solvents. However, the best results were obtained using the dichloromethane solvent. In the solvent extraction method, 8 mL of 1.0 N sodium hydroxide solution is mixed with 10‐mL dichloromethane in a separating funnel and observed as two layers, that is, one is the aqueous layer (upper layer) and second is the organic layer (lower layer), as displayed in Figure 4A. Hence, both layers were spotted on a TLC plate, and it was found that enantiomeric impurity, drug substance, and phosphate salt were in the aqueous layer, and no components were found in the organic layer by using TLC UV cabinet long wavelength. However, the aqueous layer was not suggestible to inject in normal phase chromatography as it contains phosphate salt and leads to destruction of the column. Sodium chloride was introduced to the aqueous layer to transfer the drug substance and impurity to the organic layer (Figure 4B). The presence of drug and impurity in the organic layer was confirmed by TLC, as shown in Figure 4C. Furthermore, the presence of phosphate in the aqueous layer was confirmed by the molybdenum blue method. In the molybdenum blue method, ammonium molybdate reagent is added into the separating funnel, containing both organic and aqueous layers. The change of the color of the upper layer (aqueous layer in Figure 4D) into blue confirms the complete transfer of phosphate into the aqueous layer. Figure 4E displays the images of color of the solutions of standard phosphate solution, aqueous layer, and organic layers, respectively, after the molybdenum blue test method. The blue color of the aqueous solution and colorless organic layer solution indicate phosphate salt remains in the aqueous layer. Further color of the aqueous layer is compared with standard phosphate salt solutions, and the presence of phosphate is confirmed.

### 3.3 Confirmation of Phosphate Extraction Using Phosphorus (^31^P) NMR Spectroscopy

**FIGURE 5:**
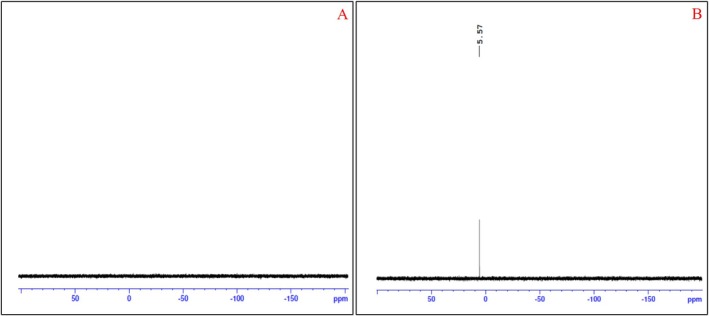
(A) ^31^P NMR spectra for organic layer. (B) ^31^P NMR spectra for aqueous layer.

^31^P NMR analysis was employed to examine the phosphorus in the aqueous and organic layers and the corresponding spectrum given in Figure 5. The NMR spectrum in Figure 5A indicates that no phosphorus peak was noticed in the organic layer, but in the aqueous layer, a phosphorous peak is identified at 5.57 ppm. The aqueous layer shows a signal at 5.57 ppm due to Na_3_PO_4_ (H_3_PO_4_ reacts with NaOH and produces trisodium phosphate) [23], as shown in Figure 5B. It indicates that the organic layer is free from phosphate salt.

### 3.4 Quantification of Phosphate Through HPLC Method

**FIGURE 6:**
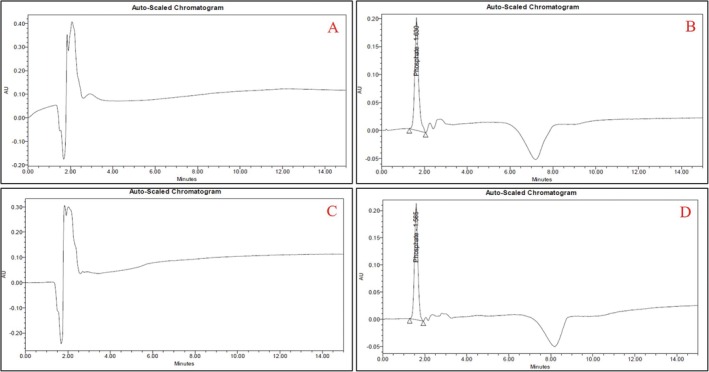
(A) Blank solution. (B) Phosphate standard solution. (C) Organic layer. (D) Aqueous layer. Standard solution contains phosphate at a concentration of 23.0%. Analytical column: ad Hoc Alpha‐C18 (150 × 4.6 mm), 3 μm; mobile phase: mixture of pH 8.2 buffer (containing 0.5 mM tetrabutylammonium hydroxide and 1 mM phthalate) and acetonitrile (95:5, %*v/v*); flow rate: 1.0 mL/min; run time: 15 min; column temperature: 25°C; injection volume: 50 μL; UV detection at 254 nm.

Reversed‐phase ion‐pair HPLC with an indirect UV detection method [24] was employed to examine the phosphate in the aqueous and organic layers and the related chromatograms provided in Figure 6. The blank chromatogram provided in Figure 6A confirms that no interference was observed at the retention time of the phosphate peak. The standard phosphate peak was found at a retention time of 1.630 min with an area of 2,450,168, as per the chromatogram shown in Figure 6B. The chromatogram of the organic layer in Figure 6C shows no phosphate peak response at RT 1.63 min. Similarly, the aqueous layer shows a peak response of 2,357,527 at RT 1.585 min due to phosphate, as shown in Figure 6D. HPLC analysis revealed that the optimized extraction procedure successfully reduced the phosphate in the organic layer, indicating that the organic layer was free from phosphate salt.

### 3.5 Method Validation

According to the ICH guidelines, validation of analytical methods as outlined in Q2 (R2) is crucial to ensure the reliability and accuracy of results in pharmaceutical analysis. System suitability ensures that the analytical system consistently provides accurate and precise results over time. Specificity tests the method's ability to differentiate the analyte from other components in the sample matrix, confirming its selectivity of the method. The limit of detection (LoD) determines the lowest concentration of the analyte. Linearity evaluates the detector response, which correlates with the analyte concentration, ensuring the method's accuracy across a range of concentrations that effectively covers the intended range of concentrations. Method precision evaluates the method's repeatability under various conditions, while accuracy measures the closeness of results with the true value.

#### 3.5.1 System Suitability/System Precision

System suitability experiments were conducted using a 0.20% standard solution, and results are summarized in Table 2. Six replicate injection responses were taken, and their values were listed alongside their repetition numbers. The average response of the six injections was calculated to be 175,963, with a standard deviation (STDEV) of 6578.146. The percent relative standard deviation (%RSD), a measure of the system precision, was determined to be 3.74%, indicating excellent repeatability and consistency in the response of the enantiomeric impurity (3S, 4S, 5R) peak. These results demonstrate that the analytical system met the acceptance criteria for system suitability, as the %RSD was well below the specified threshold of 10.0%. This ensures that the chiral HPLC method is suitable for further analysis of samples within the specified concentration range.

**TABLE 2:** Precision of 0.20% enantiomeric impurity (3S, 4S, 5R) standard solution.

Injection no.	Response
1	181,214
2	163,544
3	176,646
4	175,557
5	181,665
6	177,151
Average	175,963
STDEV	6578.146
%RSD	3.74

#### 3.5.2 Specificity Studies

**FIGURE 7:**
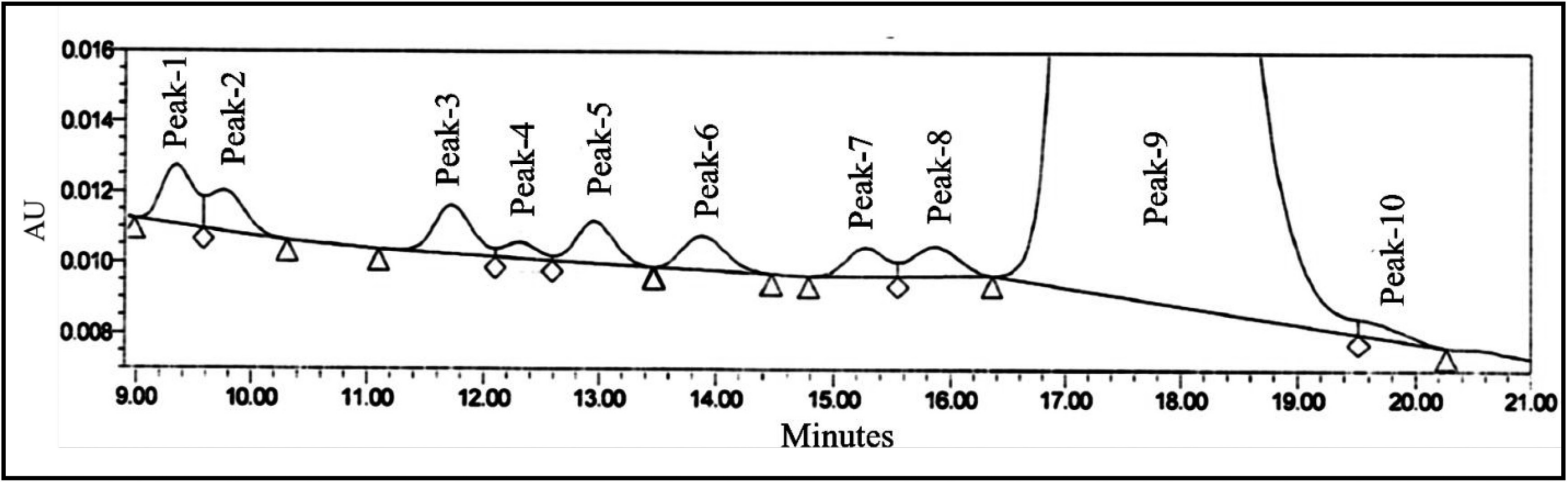
Specificity chromatogram.

Specificity was proved by preparing and injecting all impurities, including individual diastereomers and specified impurities (USP/EP monograph impurities), along with enantiomeric impurity (3S, 4S, 5R). The specificity results are summarized in Table 3, and the corresponding chromatogram is displayed in Figure 7. Here, the impurities diamine, EP impurity‐C, and EP impurity‐A were not detected, which may be due to their low UV response and less ionization in the optimized chiral method. It indicates no interference from other impurities at the retention time of enantiomeric impurity and quantifies the enantiomeric impurity in oseltamivir phosphate in the presence of other impurities. It is confirmed that this method is selective for determining enantiomeric impurity (3S, 4S, 5R) and found that the resolution between the oseltamivir peak and enantiomeric impurity is more than 3.0. The purity angle of enantiomeric impurity in the spiked sample was found to be 0.817 and a peak threshold of 1.759. The purity angle (0.715) of oseltamivir is less than the purity threshold (1.005). Oseltamivir and enantiomeric impurity satisfy the peak purity requirements, which state that the purity angle must be below the peak threshold.

**TABLE 3:** Specificity results.

Peak number	Peak name	Retention time of impurities (min)	Retention time of impurities in spiked sample (min)
Peak 1	Oseltamivir phenol impurity (EP impurity‐D)	9.318	9.348
Peak 2	(3R,4R,5R) oseltamivir	9.761	9.765
Peak 3	(3S,4S,5S) oseltamivir	11.690	11.723
Peak 4	Triphenyl phosphine oxide	12.310	12.318
Peak 5	5‐Acetyl ester impurity (EP impurity‐G)	12.952	12.958
**Peak 6**	**Enantiomeric impurity (3S, 4S, 5R)**	**13.241**	**13.886**
Peak 7	(3S,4R,5S) oseltamivir	15.251	15.270
Peak 8	(3S,4R,5R) oseltamivir	15.863	15.874
Peak 9	(3R,4S,5R) + (3R,4S,5S) + oseltamivir	17.580	17.361
Peak 10	Methyl ester impurity (EP impurity‐E)	19.667	19.565

#### 3.5.3 Linearity

**FIGURE 8:**
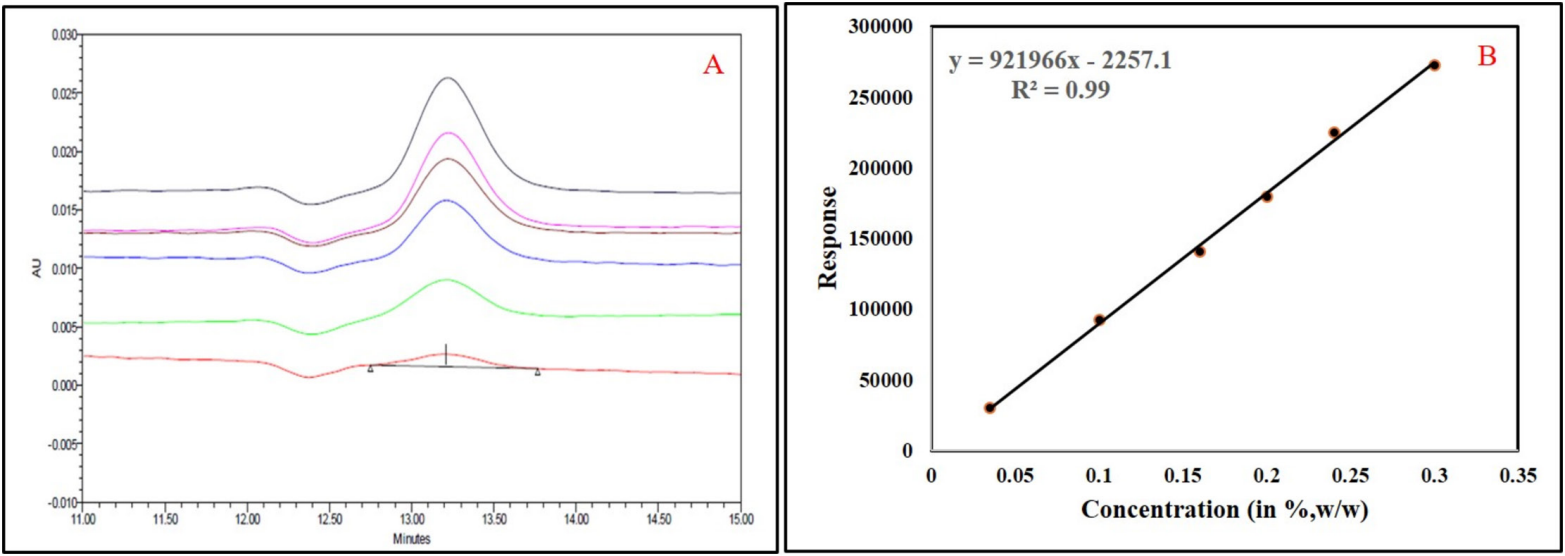
(A) Overlay chromatogram. (B) Linearity curve for the enantiomeric impurity from 0.035 to 0.300%*w/w*.

The linearity of the method was evaluated using six concentration levels of enantiomeric impurity standard solutions, ranging from 0.035 to 0.300%*w/w*. The peak response values obtained at each concentration were plotted against their respective concentrations to generate calibration curves. The results, summarized in Table S1, demonstrate a strong linear relationship between standard response and concentration, as indicated by correlation coefficients (*r* ^2^) consistently exceeding 0.99 for all concentration levels. The peak response values at each concentration level align closely with the expected values, affirming the method's ability to quantitatively determine the enantiomer within the specified range from the limit of quantification (LoQ) up to 150% of the specification limit The linearity graph (Figure 8) visually represents these findings, showing a linear trend with minimal deviation, further confirming the method's suitability for accurate and precise quantitative analysis of enantiomer in the tested range.

#### 3.5.4 Detection Limits and Precision at LoQ

**FIGURE 9:**
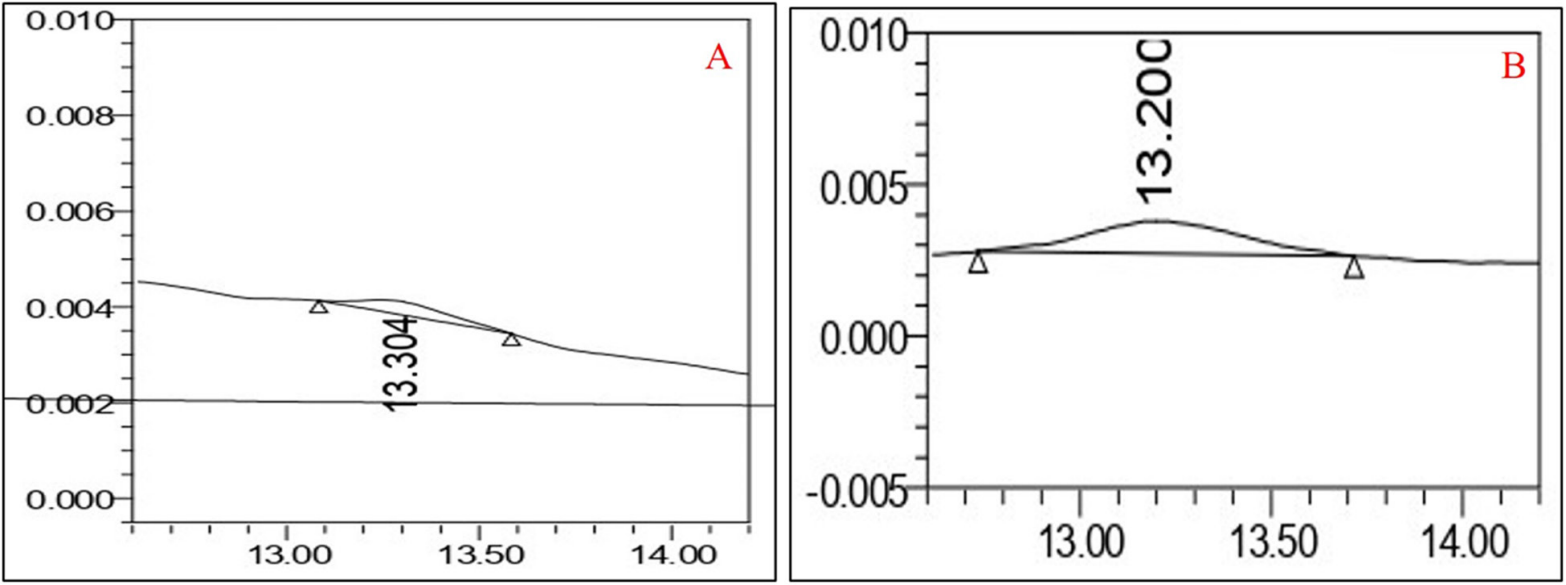
(A) Enantiomeric impurity at LoD level. (B) Enantiomeric impurity at LoQ level.

LoD and LoQ experiments were performed and found *s*/*n* value 5 at a concentration of 0.005%*w/w* having a response of 4442 and *s*/*n* value 24 at a concentration of 0.035%*w/w* having response of 30,222. The precision of the method was assessed by measuring the peak response of enantiomeric impurity at the LoQ. Table 4 summarizes the results of six replicate injections at LoQ level. The average peak response values were calculated, showing good precision with the relative standard deviation (%RSD) of 0.88%. These results demonstrate the method's ability to quantify enantiomeric impurity at low levels precisely, meeting the criteria outlined in the method validation guideline and LoD and LoQ chromatograms in Figure 9.

**TABLE 4:** Precision at LoQ levels.

Repetition no.	Response
	LoQ (0.035%*w/w*)
1	30,222
2	30,887
3	30,847
4	30,459
5	30,848
6	30,549
Average	30,635
STDV	269.3538
%RSD	0.88

#### 3.5.5 Precision (Method Precision and Intermediate Precision)

Method precision and intermediate precision were evaluated by assessing the repeatability of spiked sample solutions at the specification level of 0.20% *w/w*. Table 5 presents the results of this evaluation, where six spiked samples were analyzed to determine the method's precision and %RSD values found as 2.97%, indicating satisfactory repeatability. The intermediate precision and %RSD values were found as 1.0%, indicating satisfactory repeatability and consistency in the measurements by using different columns and different days with different analysts. Table 5 presents the results of this evaluation, where 12 spiked samples were analyzed to determine the method precision and intermediate precision and %RSD values found as 5.68%, indicating satisfactory repeatability and consistency in the measurements of method precision and intermediate precision. These findings emphasize the method's reliability and suitability for accurately quantifying enantiomeric impurity at the specified concentration level, meeting the required precision criteria outlined in the method validation protocol.

**TABLE 5:** Method precision and intermediate precision results.

Preparation	Enantiomeric impurity content (in %*w/w*)	
	Intermediate precision	Method precision
1	0.21	0.18
2	0.20	0.18
3	0.21	0.18
4	0.21	0.19
5	0.22	0.19
6	0.21	0.19
Average	0.21	0.185
STDEV	0.0000	0.0055
%RSD	0.10	2.97
Average	0.19	
STDEV	0.0108	
%RSD	5.68	

#### 3.5.6 Accuracy

The accuracy of the method was assessed through recovery studies by spiking samples at four concentration levels: LoQ, 50%, 100%, and 150% of the specification level, corresponding to concentrations of 0.035%, 0.10%, 0.20%, and 0.30%, respectively. Table 6 presents the recovery data, with three preparation responses recorded at each spiked level. The relative standard deviation (%RSD) for all spiked levels was less than 4.0%. The recovery percentages obtained ranged from 91% to 94%, indicating that the method accurately quantifies enantiomeric impurity content in oseltamivir phosphate.

**TABLE 6:** Accuracy results.

Levels	Mean % recovery (*n* = 3)	%RSD
LoQ level (0.035%, *w/w*)	93.0	3.56
50% level (0.10%, *w/w*)	91.8	2.83
100% level (0.20%, *w/w*)	93.7	0.10
150% level (0.30%, *w/w*)	92.6	0.98

#### 3.5.7 Range

The range of analytical methods was determined using data from linearity and accuracy studies. Based on these findings, the method's operational range extends from the LoQ to 150% of the specification level. This range encompasses concentrations ranging from the lowest quantification limit (0.035%*w/w*) to 1.5 times (0.30%*w/w*) of the specified threshold, ensuring the method's suitability for quantitative analysis across a broad spectrum of enantiomeric impurity content concentrations.

#### 3.5.8 Robustness

A robustness study was conducted for the determination of enantiomeric impurity in oseltamivir phosphate by chiral HPLC by varying column flow (±10%) and column temperature (±5°C) and mobile phase composition (±10% of each solvent) and found that the tailing factor is between 0.9 and 1.3 and resolution found more than 3.4 as shown in Table 7.

**TABLE 7:** Robustness results.

Robustness condition	Tailing factor	Resolution
Column flow 0.6 mL/min (as such)	1.1	3.5
Column flow 0.54 mL/min	1.3	3.5
Column flow 0.66 mL/min	0.9	3.4
Column temperature 30°C	1.0	3.8
Column temperature 40°C	1.1	3.4
n‐Hexane, methanol, isopropyl alcohol, and diethylamine (855:100:45:2) (*v/v/v/v*)	1.1	3.8
n‐Hexane, Methanol, Isopropyl alcohol and Diethylamine (845:100:55:2) (*v/v/v/v*)	1.0	3.9
n‐Hexane, methanol, isopropyl alcohol, and diethylamine (840:110:50:2) (*v/v/v/v*)	1.0	3.7
n‐Hexane, methanol, isopropyl alcohol, and diethylamine (860:90:50:2) (*v/v/v/v*)	1.1	3.8

#### 3.5.9 Solution Stability

Prepared standard solution and spiked sample solution were as described in the methodology and analyzed by keeping the solutions at 2°C–8°C at different intervals (i.e., initial, Day 1, and Day 2) and found standard and the spiked sample is stable up to 3 days, that is, more than 72 h in 2°C–8°C conditions.

### 3.6 Application of the Method

The method performance was evaluated for detecting enantiomer impurity in commercially available oseltamivir phosphate drug substances, and in that, enantiomer impurity was not detected. The developed and validated procedures embrace potential for future application in quantifying enantiomer impurities in other phosphate salt–containing drugs, such as tedizolid phosphate, tenofovir disoproxil phosphate, chloroquine phosphate, ruxolitinib phosphate, remdesivir triphosphate, sitagliptin phosphate, adenosine phosphate, and adenosine triphosphate. Here, further optimization might be required, depending on the specific drug and the matrix composition of the final pharmaceutical product.

## 4 Conclusions

The methodology presented in this work demonstrated the removal of phosphate salt using the solvent extraction method, which improved the peak shape of enantiomeric impurity. The method's sensitivity was proved by its LoD of 0.005%*w/w* and the LoQ of 0.035%*w/w* with a precision of 0.88. The linearity of the method is in the range of 0.035–0.300%*w/w* with a correlation coefficient (*r* ^2^) of 0.99. The precision was verified through system precision, method precision, and intermediate precision, and %RSD was found to be 3.74%, 2.97%, and 0.10%, respectively. % recovery of enantiomeric impurity in spiked samples was found between 91% and 94% with %RSD values below 4.0%. The robustness of the method proved and found that the resolution between impurity and drug substance was more than 3.4, and the tailing factor was less than 1.3. This innovative approach effectively addresses the challenge of quantifying enantiomeric impurity (3S, 4S, 5R) in oseltamivir phosphate drug, providing a reliable and validated method that meets international guidelines and significantly contributes to pharmaceutical quality control.

## Author Contributions

**Torati Srinivas:** conceptualization, methodology, validation, writing – original draft, investigation. **K.V.N. Suresh Reddy:** conceptualization, supervision, writing – review and editing. **Challa Madhavi:** data curation, investigation. **M. Kiranmai Reddy:** data curation.

## Supporting information


**FIGURE S1**. Reversed‐phase HPLC chromatogram for oseltamivir phosphate enantiomer content. (A) Mixture of impurities. (B) Enantiomer. (C) Oseltamivir phosphate. Analytical column: Chiralpak IE (250 × 4.6 mm), 3 μm; mobile phase: 10 mM ammonium bicarbonate:acetonitrile in the ratio of 70:30 (%*v/v*); flow rate: 0.5 mL/min; run time: 40 min; column temperature: 40°C; injection volume: 15 μL; UV detection at 220 nm.
FIGURE S2. Reversed‐phase HPLC chromatogram for oseltamivir phosphate diastereomer content. Analytical column: X‐Bridge C8 (250 × 4.6 mm), 5 μm; mobile phase: 20 mM ammonium bicarbonate:acetonitrile in the ratio of 70:30 (%*v/v*); flow rate: 0.6 mL/min; run time: 60 min; column temperature: 55°C; injection volume: 25 μL; UV detection at 220 nm.
FIGURE S3. Method development tail 1. This solution contains enantiomer at a concentration of 0.2%. Analytical column: Chiralpak IC (150 × 4.6 mm), 3 μm; mobile phase: n‐hexane:ethanol:methanol:ethanolamine in the ratio of 90:6:4:0.2 (*v/v/v/v)*; flow rate: 1.0 mL/min; run time: 60 min; column temperature: 15°C; injection volume: 20 μL; UV detection at 220 nm.
FIGURE S4. Method development tail 2. Concentration of enantiomer is 0.2%. Analytical column: Chiralpak IC (150 × 4.6 mm), 3 μm; mobile phase: n‐hexane:methanol:2‐propanol:triethlamine in the ratio of 85:10:5:0.2 (*v/v/v/v)*; flow rate: 0.6 mL/min; run time: 40 min; column temperature: 35°C; injection volume: 10 μL; UV detection at 225 nm.
FIGURE S5. Method development tail 3. Concentration of enantiomer is 0.2%. Analytical column: Chiralpak IC (150 × 4.6 mm), 3 μm; mobile phase: n‐hexane:methanol:2‐propanol:ethanolamine in the ratio of 85:10:5:0.2 (*v/v/v/v)*; flow rate: 0.6 mL/min; run time: 40 min; column temperature: 35°C; injection volume: 10 μL; UV detection at 225 nm.
TABLE S1. Linearity regression analysis.

## Data Availability

The data that support the findings of this study are available from the corresponding author upon reasonable request.
