# Supplementary material for: Quantification of Oseltamivir Phosphate Enantiomeric Impurity by Chiral HPLC Method With the Aid of Solvent Extraction and Phosphate Salt‐Out Method
Source: Chirality. 2025 May 7;37(5):e70034. doi: 10.1002/chir.70034 (PMC12058278; doi:10.1002/chir.70034)
Supplement: Supplementary file 1 — FIGURE S1. Reversed‐phase HPLC chromatogram for oseltamivir phosphate enantiomer content. (A) Mixture of impurities. (B) Enantiomer. (C) Oseltamivir phosphate. Analytical column: Chiralpak IE (250 × 4.6 mm), 3 μm; mobile phase: 10 mM ammonium bicarbonate:acetonitrile in the ratio of 70:30 (%v/v); flow rate: 0.5 mL/min; run time: 40 min; column temperature: 40°C; injection volume: 15 μL; UV detection at 220 nm. FIGURE S2. Reversed‐phase HPLC chromatogram for oseltamivir phosphate diastereomer content. Analytical column: X‐Bridge C8 (250 × 4.6 mm), 5 μm; mobile phase: 20 mM ammonium bicarbonate:acetonitrile in the ratio of 70:30 (%v/v); flow rate: 0.6 mL/min; run time: 60 min; column temperature: 55°C; injection volume: 25 μL; UV detection at 220 nm. FIGURE S3. Method development tail 1. This solution contains enantiomer at a concentration of 0.2%. Analytical column: Chiralpak IC (150 × 4.6 mm), 3 μm; mobile phase: n‐hexane:ethanol:methanol:ethanolamine in the ratio of 90:6:4:0.2 (v/v/v/v); flow rate: 1.0 mL/min; run time: 60 min; column temperature: 15°C; injection volume: 20 μL; UV detection at 220 nm. FIGURE S4. Method development tail 2. Concentration of enantiomer is 0.2%. Analytical column: Chiralpak IC (150 × 4.6 mm), 3 μm; mobile phase: n‐hexane:methanol:2‐propanol:triethlamine in the ratio of 85:10:5:0.2 (v/v/v/v); flow rate: 0.6 mL/min; run time: 40 min; column temperature: 35°C; injection volume: 10 μL; UV detection at 225 nm. FIGURE S5. Method development tail 3. Concentration of enantiomer is 0.2%. Analytical column: Chiralpak IC (150 × 4.6 mm), 3 μm; mobile phase: n‐hexane:methanol:2‐propanol:ethanolamine in the ratio of 85:10:5:0.2 (v/v/v/v); flow rate: 0.6 mL/min; run time: 40 min; column temperature: 35°C; injection volume: 10 μL; UV detection at 225 nm. TABLE S1. Linearity regression analysis. [file CHIR-37-e70034-s001.docx]

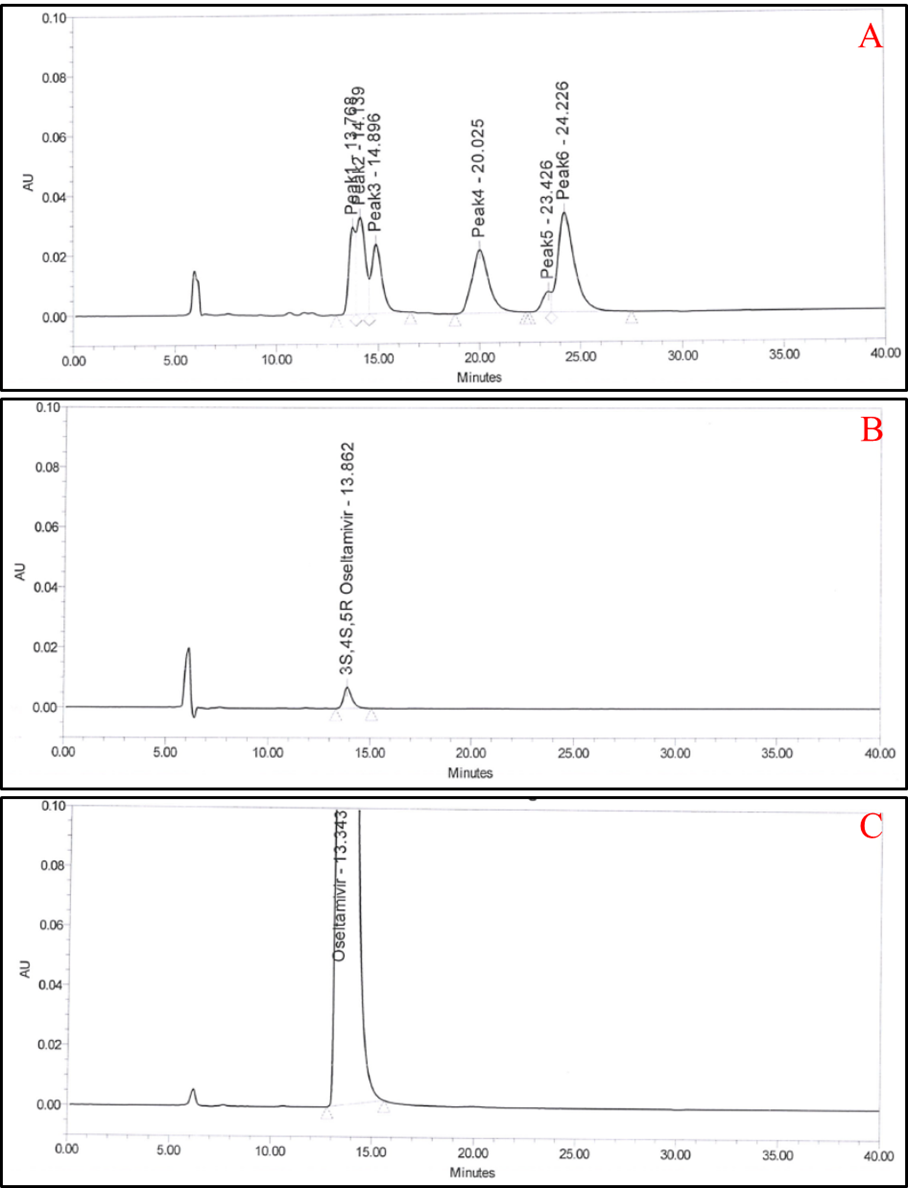


**FIGURE S1** Reverse phase HPLC chromatogram for Oseltamivir phosphate Enantiomer content. A) Mixture of impurities, B) Enantiomer, C) Oseltamivir phosphate. [Analytical column: Chiralpak IE (250 x 4.6) mm, 3 µm; mobile phase: 10mM Ammonium bicarbonate: Acetonitrile in the ratio of 70:30 (%*v/v*); flow rate: 0.5 mL/min; run time:40 min; Column temepature:40°C; injection volume :15µL; UV detection at 220 nm]

**
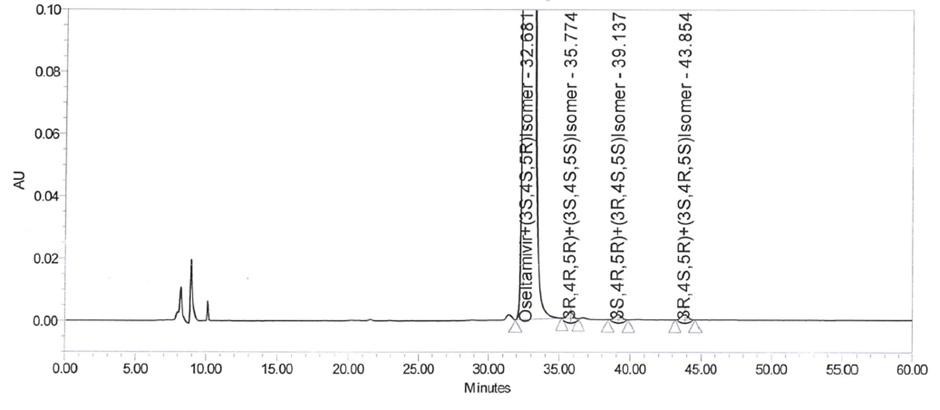
**

**FIGURE S2** Reverse phase HPLC chromatogram for Oseltamivir phosphate Diasteriomers content. [Analytical column: X-Bridge C8 (250 x 4.6) mm, 5 µm; mobile phase: 20mM Ammonium bicarbonate: Acetonitrile in the ratio of 70:30 (%*v/v*); flow rate: 0.6 mL/min; run time: 60 min; Column temperature: 55°C; injection volume: 25µL; UV detection at 220 nm]


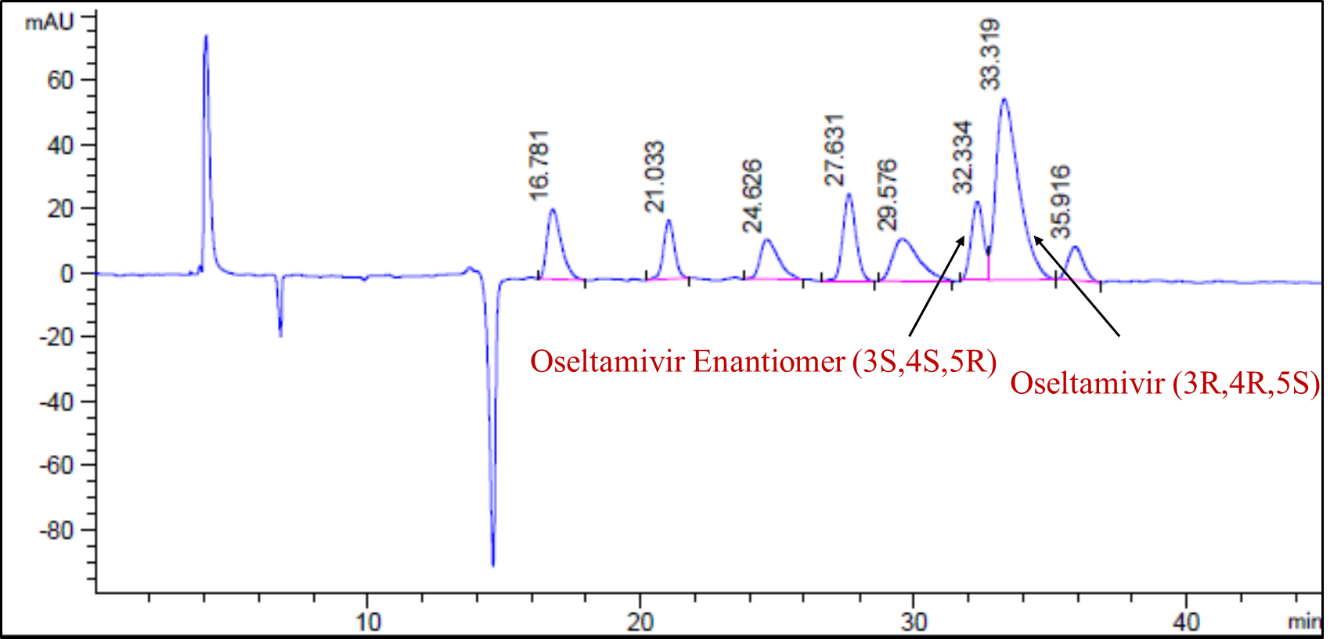


**FIGURE S3** Method development tail 1 .This solution contains Enantiomer at a concentration of 0.2%. [Analytical column: Chiralpak IC (150 x 4.6) mm, 3 µm; mobile phase: n-Hexane:Ethanol:Methanol:Ethanolamine in the ratio of 90:6:4:0.2 (*v/v/v/v)*; flow rate: 1.0 mL/min; run time:60 min; Column temepature:15°C; injection volume: 20µL; UV detection at 220 nm]

**FIGURE S4** Method development tail 2. [Concentration of Enantiomer is 0.2%. Analytical column: Chiralpak IC (150 x 4.6) mm, 3 µm; mobile phase: n-Hexane:Methanol:2-Propanol:Triethlamine in the ratio of 85:10:5:0.2 (*v/v/v/v)*; flow rate: 0.6 mL/min; run time:40 min; Column temepature:35°C; injection volume:10µL; UV detection at 225nm]

**FIGURE S5** Method development tail 3. [Concentration of Enantiomer is 0.2%. Analytical column: Chiralpak IC (150 x 4.6) mm, 3 µm; mobile phase: n-Hexane: Methanol: 2-Propanol: Ethanolamine in the ratio of 85:10:5:0.2 (*v/v/v/v)*; flow rate: 0.6 mL/min; run time: 40 min; Column temepature: 35°C; injection volume: 10µL; UV detection at 225 nm]

**TABLE S1** Linearity regression analysis

| **Level** | **Enantiomer concentration (%,w/w)** | **Response** |
| --- | --- | --- |
| LoQ | 0.035 | 30002 |
| 50% | 0.10 | 92604 |
| 80% | 0.16 | 140896 |
| 100% | 0.20 | 179272 |
| 120% | 0.24 | 225126 |
| 150% | 0.30 | 272792 |
| Correlation Coefficient | | 0.999 |
| Intercept | | -2257.08 |
| % Intercept at 100 % level | | -1.26 |
